# Revolutionary multi-omics analysis revealing prognostic signature of thyroid cancer and subsequent in vitro validation of SNAI1 in mediating thyroid cancer progression through EMT

**DOI:** 10.1007/s10238-024-01387-z

**Published:** 2024-06-13

**Authors:** Xin Jin, Chunlan Fu, Jiahui Qi, Chuanzhi Chen

**Affiliations:** 1 Present Address: Department of Breast Surgery, Zhuji Affiliated Hospital of Wenzhou Medical University, Zhuji, 311899 Zhejiang China; 2 Present Address: Department of Hematology, Zhuji Affiliated Hospital of Wenzhou Medical University, Zhuji, 311899 Zhejiang China; 3 Institute of Aging, Key Laboratory of Alzheimer’s Disease of Zhejiang Province, Wenzhou Medical University, Wenzhou, 325035 Zhejiang China; 4 Department of Thyroid Surgery, The First Affiliated Hospital of Wenzhou Medical University, Wenzhou, 325000 Zhejiang China

**Keywords:** Multi-omics analysis, Thyroid cancer, Prognostic model, SNAI1, EMT, Proliferation

## Abstract

Thyroid carcinoma (TC), the most commonly diagnosed malignancy of the endocrine system, has witnessed a significant rise in incidence over the past few decades. The integration of scRNA-seq with other sequencing approaches offers researchers a distinct perspective to explore mechanisms underlying TC progression. Therefore, it is crucial to develop a prognostic model for TC patients by utilizing a multi-omics approach. We acquired and processed transcriptomic data from the TCGA-THCA dataset, including mRNA expression profiles, lncRNA expression profiles, miRNA expression profiles, methylation chip data, gene mutation data, and clinical data. We constructed a tumor-related risk model using machine learning methods and developed a consensus machine learning-driven signature (CMLS) for accurate and stable prediction of TC patient outcomes. 2 strains of undifferentiated TC cell lines and 1 strain of PTC cell line were utilized for in vitro validation. mRNA, protein levels of hub genes, epithelial-mesenchymal transition (EMT)-associated phenotypes were detected by a series of in vitro experiments. We identified 3 molecular subtypes of TC based on integrated multi-omics clustering algorithms, which were associated with overall survival and displayed distinct molecular features. We developed a CMLS based on 28 hub genes to predict patient outcomes, and demonstrated that CMLS outperformed other prognostic models. TC patients of relatively lower CMLS score had significantly higher levels of T cells, B cells, and macrophages, indicating an immune-activated state. Fibroblasts were predominantly enriched in the high CMLS group, along with markers associated with immune suppression and evasion. We identified several drugs that could be suitable for patients with high CMLS, including Staurosporine_1034, Rapamycin_1084, gemcitabine, and topotecan. SNAI1 was elevated in both undifferentiated TC cell lines, comparing to PTC cells. Knockdown of SNAI1 reduced the cell proliferation and EMT phenotypes of undifferentiated TC cells. Our findings highlight the importance of multi-omics analysis in understanding the molecular subtypes and immune characteristics of TC, and provide a novel prognostic model and potential therapeutic targets for this disease. Moreover, we identified SNAI1 in mediating TC progression through EMT in vitro.

**Supplementary Information:**

The online version contains supplementary material available at 10.1007/s10238-024-01387-z.

## Introduction

Thyroid carcinoma (TC) is the most frequently diagnosed malignancy of the endocrine system, with an estimated 586,000 new cases and 43,600 deaths worldwide in 2020 [1]. Over the past few decades, there has been a continuous increase in the incidence of TC, while TC-related mortality rates are approximately 3 and 5 per million among men and women, respectively [2]. Among TCs of follicular origin, papillary TC (PTC) is the most common histologic subtype, accounting for 80–90% of all cases [3]. Despite a favorable prognosis for the majority of papillary thyroid cancer patients, recurrence is observed in nearly 30% of cases [4].

PTC prognosis varies significantly due to the heterogeneity of TC. Despite sharing a common origin, tumors exhibit distinct features resulting from spatial heterogeneity influenced by genetic, phenotypic, and behavioral factors [5, 6]. This heterogeneity is associated with both inter-tumor and intra-tumor variability [7], whereby cancer cells and cells within the TME collectively determine disease progression, therapy efficacy, and the likelihood of treatment resistance [8]. However, previous studies have primarily focused on tumor cells while neglecting the crucial role of the TME in TC development. Recent advancements in scRNA-seq provide an opportunity to analyze cellular heterogeneity more comprehensively and uncover the internal mechanisms driving tumor development [9]. Combining the scRNA-seq with other dimension of sequencing approaches might provide a unique perspective for researches to peek into the underlying mechanisms of the TC progression. In light of this, it is imperative to establish prognostic model for TC patients by resorting to the multi-omics manner.

In this study, we analyzed the cancer genome atlas-thyroid carcinoma (TCGA-THCA) dataset to identify prognostic genes and subtypes using multi-omics data. We identified three molecular subtypes of THCA based on integrated multi-omics clustering algorithms, which were associated with overall survival. These subtypes showed distinct molecular features, including enrichment of cancer pathways and transcriptional regulators. We developed a consensus machine learning-driven signature (CMLS) based on 28 hub genes to predict TC patient outcomes, and demonstrated that CMLS outperformed other prognostic models. We also analyzed the immune characteristics associated with CMLS and found differences in immune cell infiltration and immune therapy response between TC patients with relatively higher and lower CMLS score value. Furthermore, we analyzed drug sensitivity based on CMLS scores and identified potential drugs for TC patients with high CMLS score value. Our findings highlight the importance of multi-omics analysis in understanding the molecular subtypes and immune characteristics of TC, and provide a novel prognostic model and potential therapeutic targets for this disease. Moreover, we identified SNAI1 in mediating TC progression through EMT in vitro.

## Methods

### Origin of transcriptomic data

We selected mRNA expression profiles, lncRNA expression profiles, miRNA expression profiles, methylation chip data, gene mutation data, and corresponding clinical data (*n* = 489) from the TCGA-THCA. All data were transformed into TPM format and log2 transformed for subsequent analysis.

### Multi-omics consistency analysis and risk features generated by machine learning-based integrated methods

The detailed analysis approaches of these analysis were elaborated in the additional file 1.

### Molecular features and stability of common subtypes

We calculated the enrichment scores of multiple pathway-related gene sets, including the Hallmark gene set, and reconstructed transcriptional regulatory networks and regulatory subnetworks (RTNs) using the “RTN” [10] R package. The RTNs included 23 transcription factors related to induced/inhibited targets. We also compared the distribution of immune checkpoint genes among the subtypes and estimated the immune/stromal scores of tumor tissues using the “ESTIMATE” [11] R package. We calculated the tumor-infiltrating lymphocyte DNA methylation (MeTIL) scores based on specific gene sets. The enrichment of 22 tumor immune microenvironment cell types was evaluated using the CIBERSORT software [12]. For subtype stability, we compared the consensus clustering consistency with NTP and PAM classifiers.

### Construction of tumor-related risk features

We used the “MOVICS” package [13] to select mRNA as the prognostic gene set and screened for prognosis-related genes using univariate Cox analysis (*P* < 0.05). We established a prognostic model using 101 machine learning methods. The algorithm was then used to provide a CMLS score value in this manner. The TCGA cohorts were divided into TC patients of relatively higher and lower CMLS score value based on the median value of the CMLS value, and the prediction differences between the two groups were studied, along with an evaluation of the model’s accuracy.

### Differential gene analysis

Differential gene calculation was performed using the “limma” [14] package to investigate gene expression differences between TC patients of relatively higher and lower CMLS score. The results of enrichment analysis were visualized using the “enrichplot” [15] package.

### Prediction of immunotherapy response

The prediction of immunotherapy response collected data from the Braun (renal cell carcinoma, RCC), GSE91061 (melanoma), IMvigor210 (urothelial carcinoma, UC), and GSE78220 (melanoma) datasets. The risk model scores were calculated for each dataset to predict immunotherapy response.

### Tumor immune infiltration analysis and TIP analysis

We used the “IOBR” [16] package to determine the degree of immune infiltration in TCGA-THCA patients using the results of six evaluation methods, including EPIC and Estimate, etc. Box plots were created to compare immune cell infiltration in the tumor microenvironment between the two groups.

### Drug sensitivity and mutation analysis

We used the R package “oncoPredict” [17] to calculate the IC50 of common chemotherapy drugs from the GDSCv2 database and the AUC value from the CTRP dataset to evaluate the relationship between CMLS score value and drug sensitivity. Wilcoxon rank-sum test was used to compare IC50 or AUC values between the two risk groups. Mutation data for thyroid cancer were downloaded from the TCGA GDC database, and the “maftools” package [18] was used for analysis.

### Cell culture

Two strains of undifferentiated TC cell lines (C643, HTH74) and 1 strain of PTC cell line (TPC1) were obtained from ATCC. All 4 cell lines were cultured in RPMI-1640 medium supplemented with FBS. Transfection was performed using lipofectamine 3000 (invitrogen) [19]. Cell inoculation was carried out using 6-well plates.

### RNA procurement and RT-qPCR analysis

Total RNA extraction was performed following previously described protocols [20]. Reverse transcription of RNA into cDNA was conducted using a reverse transcription kit (Yeason). For subsequent PCR analysis, ChemoHS qPCR Mix was utilized along with ACTIN as a reference gene and specific primers.

### Protein preparation and western blotting (WB)

WB was performed as preciously described [21]. Cell lysate was incubated on ice for 15 min, followed by centrifugation at 20,000 × g for 30 min at 4 °C. Protein concentrations were determined using the BCA Protein Assay Kit. The proteins were then transferred onto a polyvinylidene fluoride membrane. Primary antibody: a-SMA (1:1000, EPR18430, abcam), vimentin (1:1000, EPR3776, abcam).

### Cell viability analysis, colony formation assay and transwell analysis

Cell viability was evaluated using the CCK-8 assay as preciously described [20]. Briefly, 4 × 10^3^ cells per well were seeded in 96-well microplates. 10 μL of CCK-8 reagent was added to each well. After 1 h of incubation, absorbance was measured at 450 nm. For the colony formation assay, cells were seeded in six-well plates and incubated at 36.7 °C for 11 days. To evaluate the invasion ability of TC cells, 1 × 10^5^ cells were suspended in 200 μL of DMEM medium with 5% FBS and plated into the upper chamber of a Transwell system (Corning, NY, USA) coated with matrigel. The lower chamber was filled with medium containing 20% FBS. The cells were then cultured for 24 h.

### Statistical analysis

All data processing, statistical analysis, and plotting were performed using R 4.1.3 software. Pearson correlation coefficient was used to evaluate the correlation between two continuous variables. Chi-square test was used to compare categorical variables, and Wilcoxon rank-sum test was used to compare continuous variables. Cox regression and Kaplan–Meier analysis were performed using the survival package.

## Results

### Multi-omics consensus prognostic molecular subtypes of THCA

**Fig. 1:**
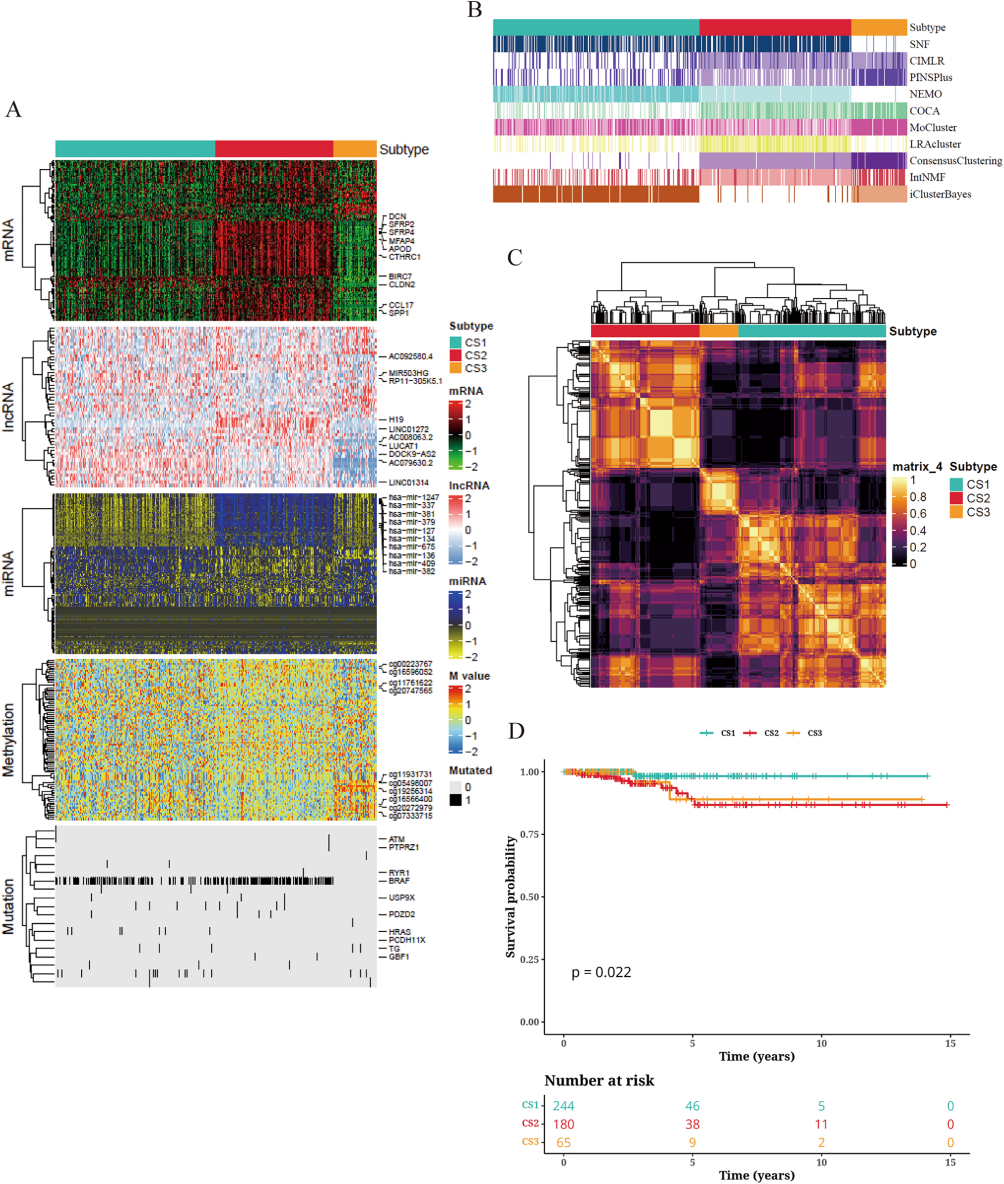
Prognostic molecular subtypes of THCA based on multi-omics consensus analysis. **A** Consensus heatmap of the integrated subtypes based on marker genes, including mRNA, lncRNA, miRNA, DNA CpG methylation sites, and mutated genes. **B** Clustering of THCA patients using 10 state-of-the-art multi-omics clustering methods. **C** Consensus clustering matrices of the three novel prognostic subtypes based on the 10 algorithms. **D** Survival analysis curves of the three subtypes in the cohort of THCA patients, shown in the form of KM curves

We independently identified three subtypes from the 10 integrated multi-omics clustering algorithms, taking into account the cluster prediction index, gap statistics analysis, silhouette scores, and previous research experience to determine the number of subtypes. Subsequently, we further integrated the clustering results with the different molecular expression patterns across transcriptomics, epigenetic methylation, and somatic cell mutation (Fig. 1A–C). Our classification system was closely associated with overall survival (OS) (*P* = 0.022; Fig. 1D). Notably, Cancer Subtype 1 (CS1) exhibited the most favorable survival outcome among all evaluated subtypes.

### Division and molecular features of integrated consensus molecular subtypes in THCA

**Fig. 2:**
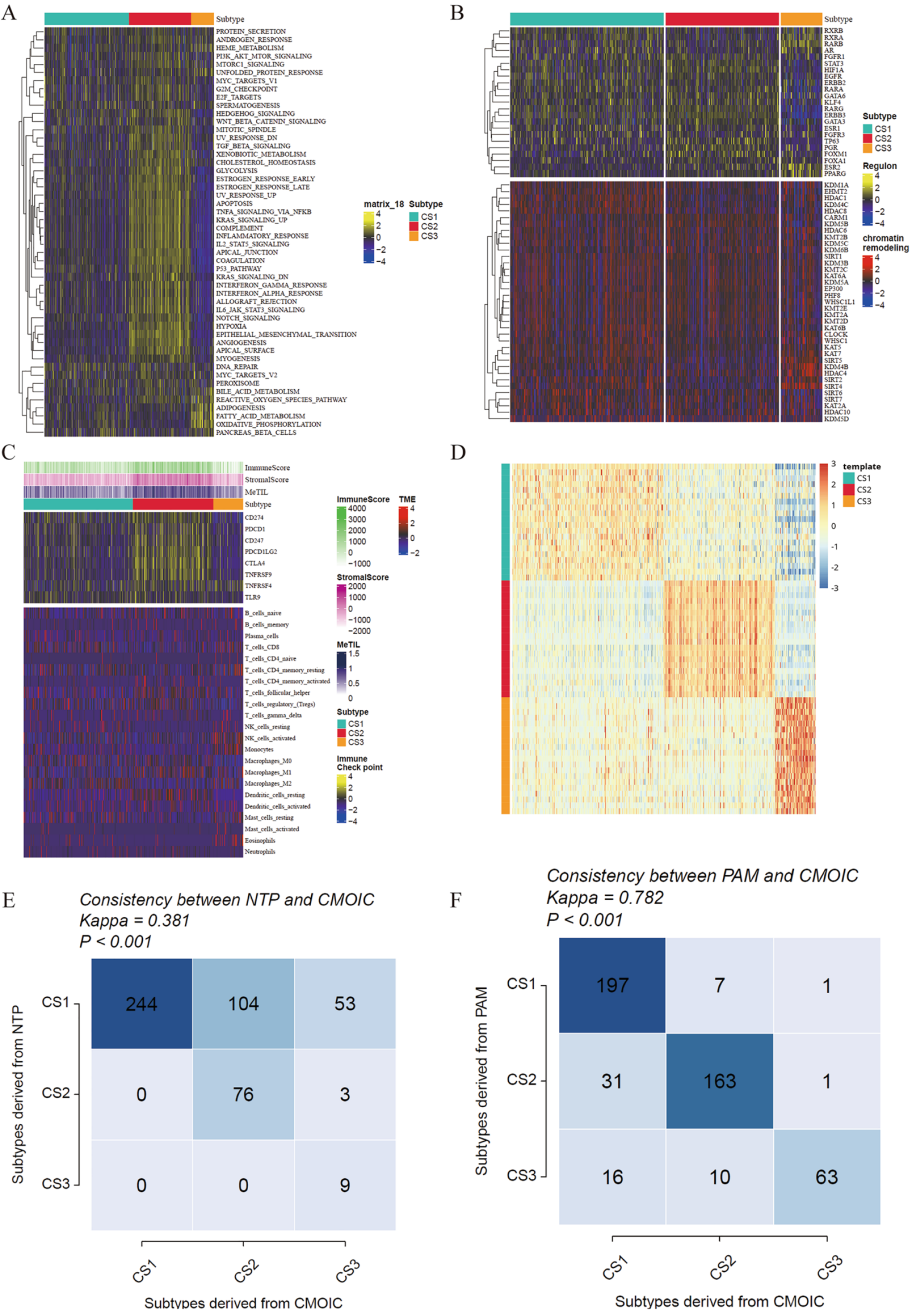
Division and molecular features of integrated consensus molecular subtypes in THCA. **A** Enrichment heatmap of hallmark cancer-related features in the three subtypes. **B** Activity profiles of 23 TFs (top) and potential regulatory factors associated with chromatin remodeling in the three subtypes (bottom). **C** Immune features in the TCGA cohort. The top annotations in the heatmap show immune enrichment scores of tumor-infiltrating lymphocytes, stromal enrichment scores, and MeTIL. The top panel shows the expression of typical immune checkpoint genes, and the bottom panel shows the enrichment levels of 22 TME-related immune cells. **D** Validation of CS in the NTP algorithm in the TCGA cohort. **E** Consistency of CS with NTP in the TCGA cohort. **F** Consistency of CS with PAM in the TCGA cohort

Currently, the molecular subtypes of THCA are largely based on the classification of molecular expression levels, which may be associated with specific biological functions. Therefore, we also attempted to explore the distinct molecular features of these CSs. Interestingly, we found that multiple cancer pathways such as hypoxia and epithelial-mesenchymal transition (EMT) were significantly enriched in CS2, while pathways such as lipid metabolism and adipogenesis were significantly enriched in CS3, consistent with an inferior survival outcome for CS2 and CS3 compared to CS1 (Fig. 2A). To further investigate transcriptional differences, we analyzed potential regulators associated with cancer chromatin remodeling and 23 transcription factors (TFs) (Fig. 2B). Regulators such as FGFR1 and TP63 were significantly activated in CS1 and CS2, while RARB, AR, ESR2, and PPARG were specifically enriched in CS3. The regulatory subnetwork activity spectrum associated with cancer chromatin remodeling further highlighted potential patterns of differential regulation among the CSs, suggesting an epigenetic-driven transcriptional network in TC progression. Given the crucial role of tumor immunity in tumor occurrence and progression, we quantified the infiltration levels of immune cells and demonstrated that immune cell infiltration was significantly increased in CS2 and CS3 but relatively lower in CS1 (Fig. 2C). Based on the results of differential expression analysis between subtypes, we selected 20 genes specifically up-regulated in each subtype as classifiers to further validate the stability of the subtypes (Fig. 2D). The consistency of CSs with the NTP and partitioning around medoids (PAM) algorithms was also assessed (*P* < 0.005; Fig. 2E–F).

### Development of CMLS prognostic model

**Fig. 3:**
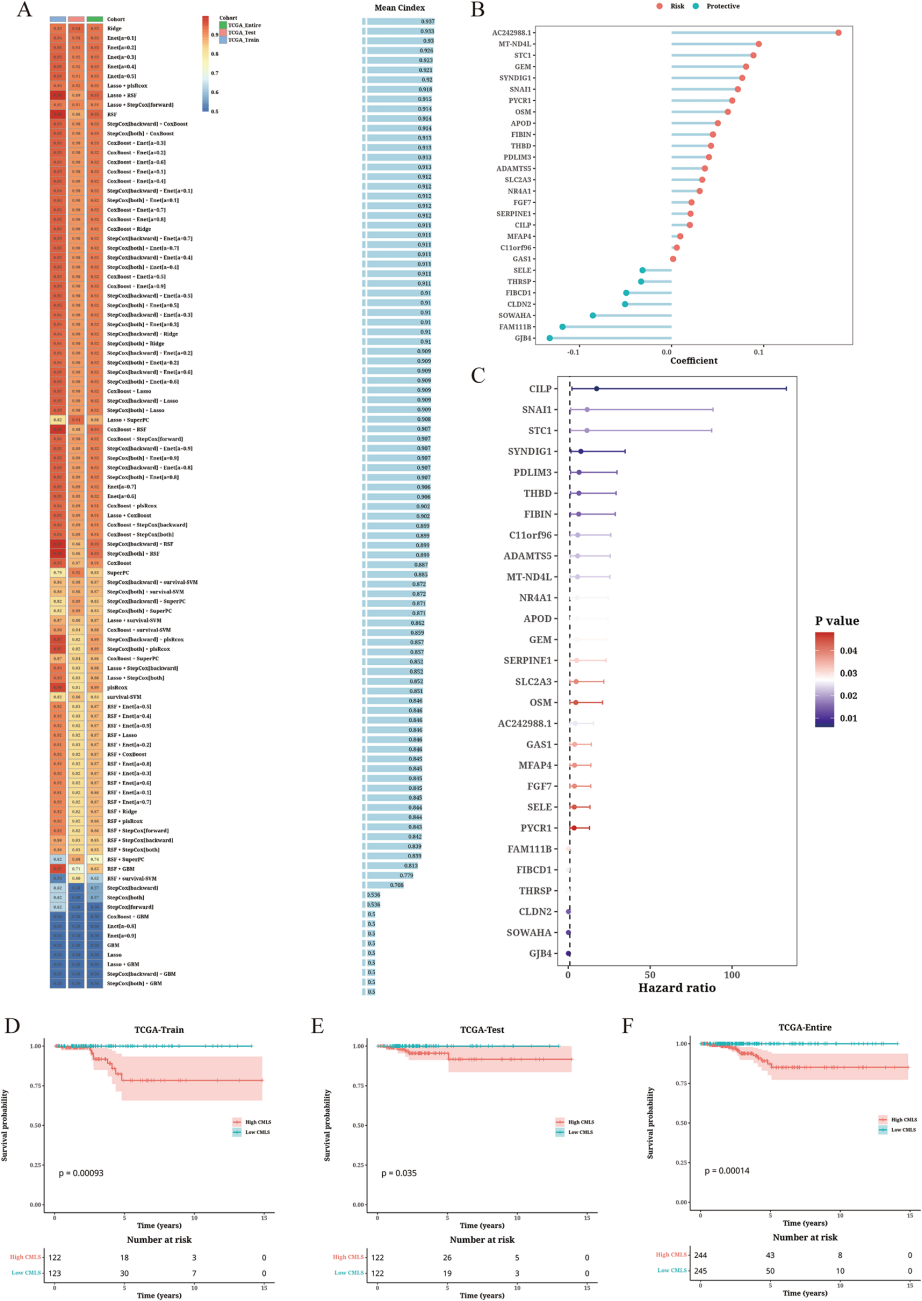
Development and validation of CMLS prognostic model in TCGA cohort. **A** Generation of 101 combinations of machine learning algorithms using a comprehensive computational framework. Calculation of C-index using the TCGA-Train, TCGA-Test, and TCGA-Entire cohorts, and ranking based on the average C-index of the validation set. **B** The identified 28 hub genes selected by the Ridge algorithm. **C** Univariate Cox regression analysis of the identified 28 hub genes in the TC training cohort. **D**–**F** Survival analysis of TC patients of different levels of CMLS scores in the TCGA-Train (**D**), TCGA-Test (**E**), and TCGA-Entire cohorts (**F**)

We identified 28 mRNA genes that were significantly associated with OS from the “MOVICS” package [22]. Subsequently, they were included in the integrated framework to execute CMLS. In the TCGA-Train training cohort, we built a consistent model based on 101 algorithm combinations and evaluated the predictive capability of all models (Fig. 3A). Out of the 101 models, the Ridge algorithm maintained the highest average C-index to construct the final model. The model was constructed with 28 hub genes (Fig. 3B, C), among which cartilage intermediate layer protein (CILP) was found with highest hazard ratio and lowest p value. We then calculated the CMLS scores for each sample in all cohorts. High CMLS patients had poorer OS in the TCGA-Train (*P* = 0.00093), TCGA-Test (*P* = 0.035), and TCGA-Entire cohorts (*P* = 0.00014) (Fig. 3D–F).

### Comparison of CMLS model with other prognostic models

**Fig. 4:**
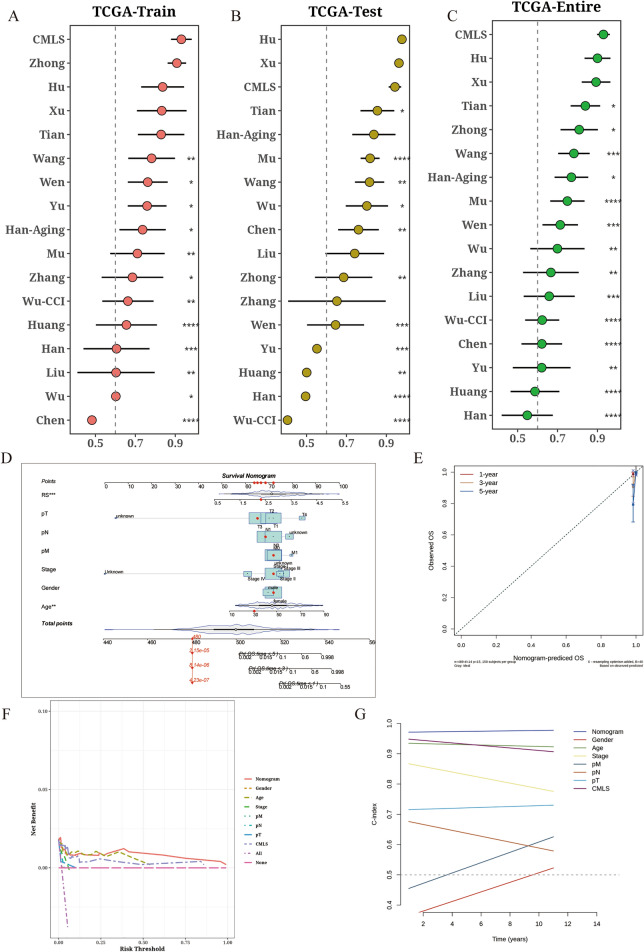
Comparison of CMLS with other prognostic models. **A**–**C** Comparison of CMLS with 16 other published TC models in the TCGA-Train, TCGA-Test, and TCGA-Entire cohorts. **D** Comprehensive column line graph constructed based on CMLS. **E** Calibration curve of the comprehensive column line graph. **F** DCA analysis demonstrating the benefit of the comprehensive column line graph in clinical practice for patients. **G** Comparison of C-index over time between the comprehensive column line graph and CMLS

To comprehensively compare CMLS with other TC prognostic model signatures, we conducted a literature search of relevant publications from the past five years and ultimately included 16 different signatures. Importantly, CMLS demonstrated better performance in terms of C-index compared to almost all models in the TCGA-Train, TCGA-Test, and TCGA-Entire datasets (Fig. 4A–C). We constructed a comprehensive nomogram and found that age and CMLS risk score value were significantly associated with prognosis (Fig. 4D). Calibration curves demonstrated the accuracy of the nomogram in predicting survival outcomes (Fig. 4E). DCA indicated that the nomogram provided greater clinical benefit for patients compared to using CMLS alone (Fig. 4F), and time-dependent C-index further confirmed the superior predictive performance of the nomogram (Fig. 4G).

### Immune characteristics associated with CMLS

**Fig. 5:**
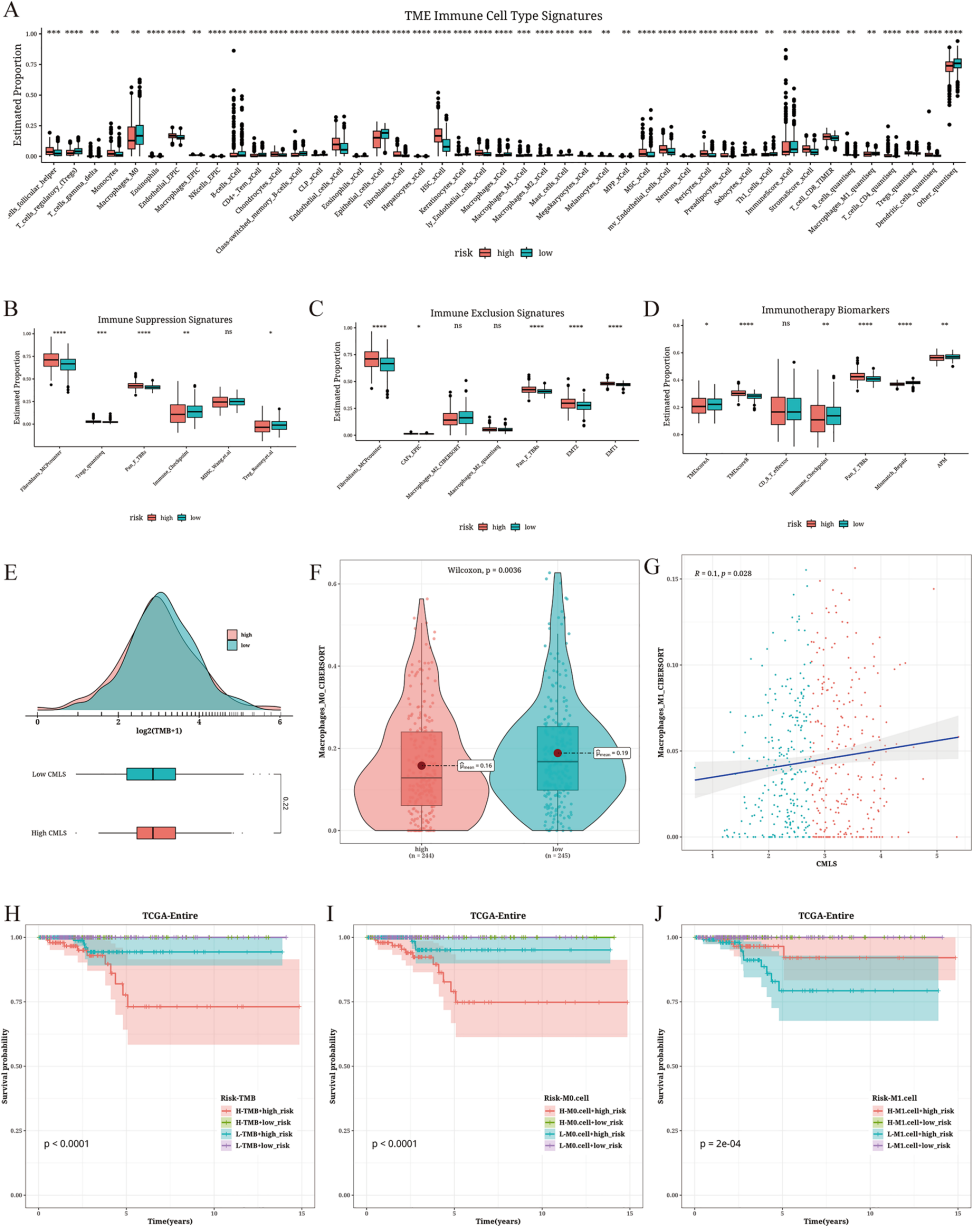
Results of immune characteristics related to CMLS. **A** Distribution of TME immune cell type features between TC patients of relatively higher and lower CMLS score value. **B** Distribution of immune suppression features between TC patients of relatively higher and lower CMLS score value. **C** Distribution of immune exclusion features between TC patients of relatively higher and lower CMLS score value. **D** Distribution of immune therapy biomarkers between TC patients of relatively higher and lower CMLS score value. **E** Distribution of TMB between TC patients of relatively higher and lower CMLS score value. **F** The abundance of M0 macrophages in the TC patients of relatively higher and lower CMLS value. **G** Correlation analysis between M1 macrophages infiltrated abundance and TC patients CMLS score value. **H**–**J** Survival analysis of TC patients in the TCGA entire cohort divided by the combination of CMLS score value with TMB (**H**), M0 macrophages (**I**), and M1 macrophages (**J**)

TC patients of relatively lower CMLS score had significantly higher levels of immune cell infiltration, including T cells, B cells, and macrophages, indicating an immune-activated state (Fig. 5A). Fibroblasts were predominantly enriched in the TC patients of relatively higher CMLS score, while molecular markers associated with immune suppression and evasion, such as the EMT pathway, were also mainly enriched in this group of TC patients, indicating an immune-suppressive state (Fig. 5B, C). Features associated with better response to immune therapy, such as TMEscoreA, immune checkpoints, mismatch repair, and antigen presentation machinery (APM), were significantly enriched in the TC patients of relatively lower CMLS score, while TMEscoreA and Pan_F_TBRs were significantly enriched in the TC patients of relatively higher CMLS score (Fig. 5D). Tumor mutation burden (TMB), a recognized biomarker for assessing patient response to immune therapy, and M0 and M1 macrophages, which play a specific role in immune therapy for bladder cancer, were also analyzed for differences in their levels between the TC patients of different CMLS score value. TC patients of relatively lower CMLS score value had lower TMB and higher M0 macrophages, while the high CMLS group exhibited the opposite pattern. Correlation analysis showed a positive correlation between M1 macrophages and CMLS score value (Fig. 5E–G). Survival analysis also revealed that CMLS could serve as an effective complementary factor to TMB, M0 macrophages, and M1 macrophages in distinguishing TC patient prognosis (Fig. 5H–J). In the TC patients of relatively higher CMLS score, lower TMB, lower M0 macrophages, or higher M1 macrophage infiltration were associated with better survival prognosis in TC patients.

### Immunotherapy analysis based on CMLS score value

**Fig. 6:**
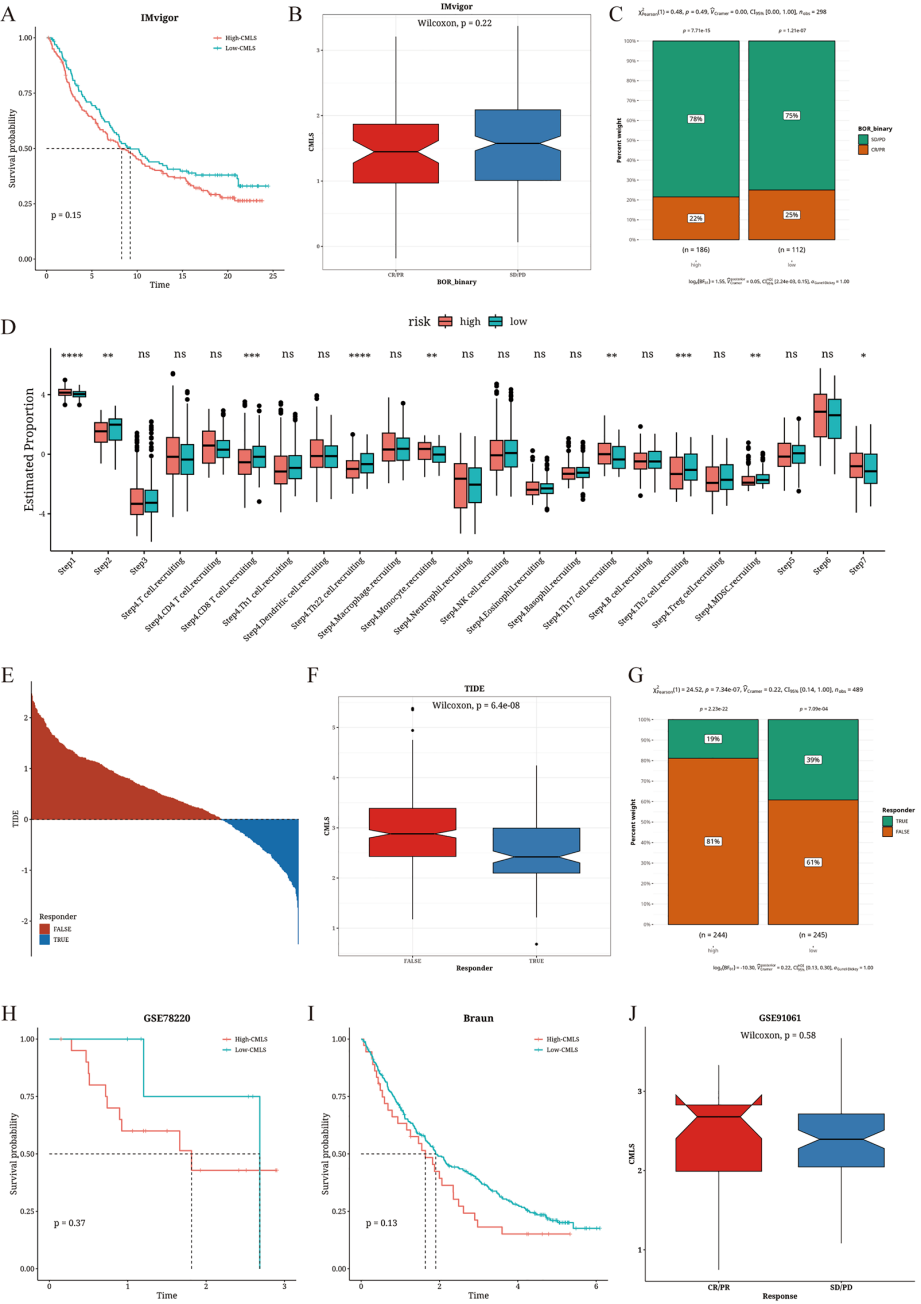
Results of immune therapy analysis based on CMLS score value. **A** Survival analysis results of TC patients of different levels of CMLS scores in the IMvigor cohort. **B** Box plot of CMLS in the Response and NonResponse groups in the IMvigor cohort. **C** Composition plot of the Response and NonResponse groups in the IMvigor cohort. **D** Differences in activation levels between TC patients of different levels of CMLS scores at each step of TIP. **E** Prediction of response to immune therapy in the TC patients of different levels of CMLS scores using the TIDE algorithm. **F** Differences in CMLS predicted by the TIDE algorithm between the TC patients of different levels of CMLS scores. **G** Differences in composition predicted by the TIDE algorithm between the TC patients of different levels of CMLS scores. **H** Survival analysis of TC patients of different levels of CMLS scores in the GSE78220 cohort. **I** Survival analysis of the high and low CMLS groups in the Braun cohort. **J** Distribution of CMLS in different immune therapy response groups in the GSE91061 cohort

We used the IMvigor dataset and calculated its CMLS value, revealing that patients with high CMLS values had worse prognosis (Fig. 6A). Boxplot results of CMLS showed that the Response group had lower CMLS values compared to the NonResponse group (Fig. 6B). As regard to the composition of the two CMLS groups, TC patients of relatively lower CMLS score had more Response patients (Fig. 6C). Subsequently, we found significant differences in immune processes, such as step1, step2, and step4 of CD8 T cell recruiting process, between TC patients of different CMLS score value (Fig. 6D). The TC patients of relatively lower CMLS score showed better responsiveness in TIDE analysis (Fig. 6E–G). We then calculated the CMLS values using three other immune datasets, namely Braun (renal cell carcinoma, RCC), GSE91061 (melanoma), and GSE78220 (melanoma), and found that patients in the high CMLS group also displayed worse prognosis (Fig. 6H, I). In the GSE91061 dataset, the Response group had higher CMLS values than the NonResponse group, although not significant (Fig. 6J).

### Drug sensitivity analysis based on CMLS score value

**Fig. 7:**
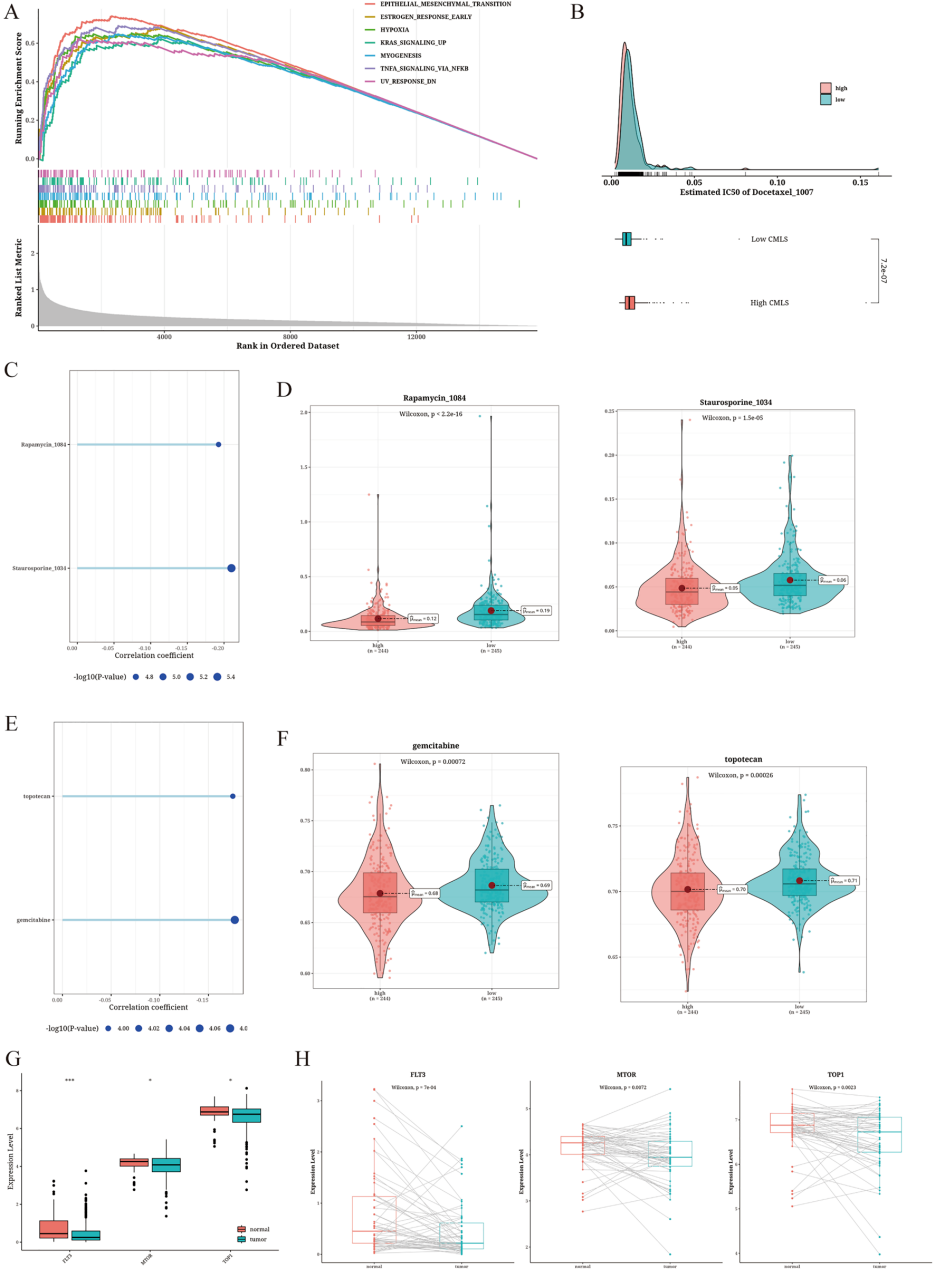
Results of drug sensitivity analysis. **A** GSEA plot of differential genes in the TC patients of relatively higher levels of CMLS scores (based on the hallmark gene set). **B** Differences in IC50 of Docetaxel_1007 between TC patients of different levels of CMLS scores. **C**–**D** Correlation and significance results of Staurosporine_1034 and Rapamycin_1084 in the GDSCv2 database. **E**, **F** Correlation and significance results of gemcitabine and topotecan in the CTRP database. **G** Differential analysis results of drug target genes FLT3 (staurosporine), KDR (rapamycin), and TOP1 (topotecan) between tumor and adjacent tissues. **H** Paired differential analysis results of drug target genes FLT3 (staurosporine), KDR (rapamycin), and TOP1 (topotecan) between tumor and adjacent tissues

GSEA showed significant activation of EMT, hypoxia, and other pathways in TC patients of relatively higher CMLS score value (Fig. 7A). We found that Docetaxel_1007 showed significant differences between the TC patients of different CMLS score value (Fig. 7B). Additionally, while focusing on significance, we also calculated the correlation with CMLS and identified several negatively correlated drugs that could be suitable for patients with high CMLS. From the GDSCv2 database, we selected Staurosporine_1034 and Rapamycin_1084, which showed significant correlation and significance (Fig. 7C, D). From the CTRP database, we selected gemcitabine and topotecan, which showed significant correlation and significance (Fig. 7E, F). Moreover, we examined the expression differences of target genes FLT3 (staurosporine), KDR (Rapamycin), and TOP1 (topotecan) between tumor and adjacent tissues (Fig. 7G, H), all of which displayed significant differences.

### Knockdown of SNAI1 significantly reduced the TC proliferation and EMT phenotypes

**Fig. 8:**
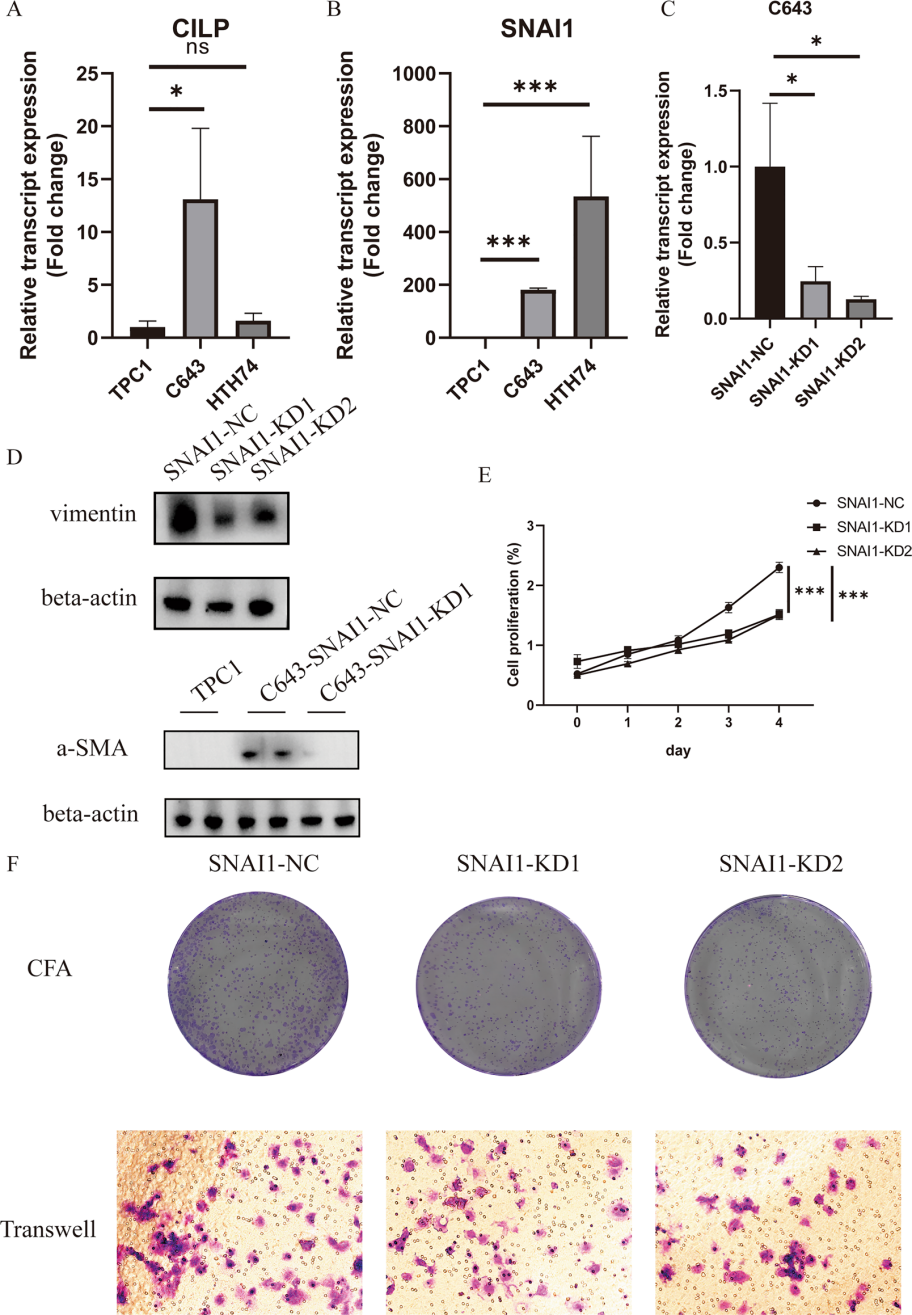
The knockdown of SNAI1 significantly reduced the TC proliferation and EMT phenotypes. **A** The mRNA transcript level of CILP in the 2 strains of undifferentiated TC cells (C643, HTH74) and 1 strain of PTC cell line (TPC1). **B** The mRNA transcript level of SNAI1 in the both strains of undifferentiated TC cells (C643, HTH74) and 1 strain of PTC cell line (TPC1). **C** The RT-qPCR validation of the knockdown of the target SNAI1 in the C643 cell line. **D** WB analysis showed up-regulation of EMT markers, including a-SMA and vimentin, in the C643 cell line with SNAI1 knockdown. **E** The CCK-8 analysis showed distinct reduction in cell proliferation in the C643 cell line with SNAI1 knockdown. **F** The CFA and transwell analysis results displaying the reduction in the colony formation capability and invasiveness of the C643 cell line with SNAI1 knockdown

We investigated the transcript levels of the hub genes derived from the CMLS model with top hazard ratio. The significant elevation of the CILP was not consistent in the 2 stains of undifferentiated TC cell lines (Fig. 8A). However, a distinguishable up-regulation of SNAI1 was observed in both 2 strains, comparing to the PTC cell line (Fig. 8B). We knocked down SNAI1 in the C643 cell line (Fig. 8C). The E-cadherin was found to be significantly reduced in the C643 cell line, comparing to both C643 cell line with SNAI1 knockdown and PTC cell line. The a-SMA and vimentin, both mesenchymal markers, were up-regulated in the C643 cell line (Fig. 8D). A significant reduction in cell proliferation rate could be found in the C643 cell line with SNAI1 knockdown (Fig. 8E). Consistent with the CCK-8 analysis results, we observed a marked reduction in the colony formation capability and invasiveness of the C643 cell line with SNAI1 knockdown (Fig. 8F). Collectively, these results suggested SNAI1 functioned as a critical promoter in TC progression through promoting EMT and cell proliferation.

## Discussion

Employing a suite of multi-omics clustering algorithms, we delineated three molecular subtypes of thyroid cancer (TC), each linked to distinct survival rates and characterized by unique molecular profiles. We developed a prognostic model named the Consensus Machine Learning-Driven Signature (CMLS), derived from the expression of 28 pivotal genes. Comparative assessments demonstrated that the CMLS model surpasses existing prognostic tools in accurately predicting outcomes for TC patients. Notably, lower CMLS scores correlated with increased levels of T cells, B cells, and macrophages, signifying an immune-active condition. Conversely, the high CMLS score subgroup was marked by an abundance of fibroblasts and exhibited profiles typical of immune suppression, which may facilitate immune evasion. Additionally, our findings suggest that drugs such as Staurosporine_1034, Rapamycin_1084, gemcitabine, and topotecan could be particularly efficacious for patients with elevated CMLS scores. We also uncovered a role for the transcription factor SNAI1 in promoting TC progression via epithelial-to-mesenchymal transition (EMT) in vitro, pointing to its potential as a novel target for therapeutic intervention.

Our CMLS model was constructed with 28 hub genes, among which cartilage intermediate layer protein (CILP) was found with highest hazard ratio and lowest p value. However, the role of CILP in the cancer progression remains largely controversial. Low CILP expression was associated with a poor prognosis in stage III-IV breast cancer. CILP was downregulated in breast cancer brain metastases (BCBM) and negatively correlated with VEGFA, which is involved in brain metastasis development. In vitro experiments showed that CILP inhibited breast cancer cell proliferation and metastasis. Further analysis revealed that CILP was associated with immune effects and T cell receptor signaling in BCBM [23]. In the context of esophageal squamous cell carcinoma (ESCC), 7 stemness-related genes, including CILP, were found to be associated with prognosis, gene mutations, and immune cell infiltration in ESCC. Knockdown of CILP expressions with siRNAs largely inhibited the proliferation of KYSE150 cells [24]. These results suggested that further research into CILP was warranted.

We observed that TC patients of relatively lower CMLS score had significantly higher levels of infiltration of T cells, indicating an immune-activated state. The density of lymphocytes in PTC has been found to be associated with improved overall survival and lower recurrence rates [25]. Proliferating lymphocytes, identified by their expression of the nuclear antigen Ki-67, have been shown to predict enhanced disease-free survival in children and young adults with PTC [26]. Infiltration of CD8+ T cells in TCs has also been associated with improved disease-free survival and reduced tumor sizes [27]. IL-2 and IL-15 play a role in regulating the expression of cytolytic proteins that are involved in T cell cytotoxicity. Treatment strategies that induce overexpression of IL-2/IL-15 in the tumor microenvironment of TCs could potentially activate T cells with cytotoxic activity. Alternative delivery methods, such as encoding IL-2 in an oncolytic virus, are being explored [28]. In their study, the researchers investigated the presence of immune markers in advanced thyroid cancer patients, evaluating their viability as therapeutic targets. The analysis consistently detected high levels of immune markers including PD-L1, FoxP3, PD-1, and CD8 within the cancerous tissues, indicating a significant role for these markers in both the progression and potential treatment of the disease. Immunohistochemistry revealed the presence of CD8( +) and FoxP3( +) T cells in all advanced tumors and some metastases. PD-1( +) lymphocytes were found in 50% of thyroid cancers. The study suggests that PD-1 checkpoint blockades could be effective in treating aggressive forms of thyroid cancer [29].

Markers associated with immune suppression and evasion, in particular with EMT pathway, were also mainly enriched in TC patients with higher CMLS score, indicating an immune-suppressive state. A mechanism study revealed that N-cadherin was associated with EMT in TC [30]. Another marker of EMT, CDH16, was found to be negatively expressed and declined to a greater extent than E-cadherin, regardless of its positive or negative expression [31]. EMT was identified as a feature of TC patients with high CMLS score. Detailed analysis investigating whether the hub genes were implicated in the TC EMT progression could shed some light on this direction, leading to a better understanding of TC carcinogenesis. In our study, we found that SNAI1 was elevated in both undifferentiated TC cell lines, comparing to PTC cells. Knockdown of SNAI1 reduced the cell proliferation and EMT phenotypes of undifferentiated TC cells. The researchers found that miR-199a-5p expression was reduced and SNAI1 expression was increased in PTC tissues and cells. They showed that overexpression of miR-199a-5p and knockdown of SNAI1 inhibited invasion and EMT of PTC cells in vitro. Furthermore, they found that miR-199a-5p directly targeted SNAI1 and downregulated its expression in PTC cells [32]. These results were consistent with our observation that SNAI1 was up-regulated in the undifferentiated TC cells, which was characterized with an inferior prognosis. We found SNAI1 as a pivotal regulator of the EMT in the undifferentiated TC cells, while more detailed analysis targeting the underlying molecular mechanism warranted further investigation.

## Conclusion

Our study emphasizes the application of multi-omics analysis in unraveling the molecular subtypes and immune characteristics of TC. We have developed a novel prognostic model and identified potential therapeutic targets for this disease. Moreover, we identified SNAI1 in mediating TC progression through EMT in vitro.

## Supplementary Information

Below is the link to the electronic supplementary material.

Supplementary file1 (DOCX 16 KB)

## Data Availability

All raw data are available through the corresponding author.
