# Supplementary material for: Revolutionary multi-omics analysis revealing prognostic signature of thyroid cancer and subsequent in vitro validation of SNAI1 in mediating thyroid cancer progression through EMT
Source: Clin Exp Med. 2024 Jun 13;24(1):127. doi: 10.1007/s10238-024-01387-z (PMC11176101; doi:10.1007/s10238-024-01387-z)
Supplement: Supplementary file 1 — Supplementary file1 (DOCX 16 KB) [file 10238_2024_1387_MOESM1_ESM.docx]

**Multi-Omics Consistency Analysis**

We utilized the "getElites" function from the MOVICS package for gene feature selection. For continuous variables (mRNA, lncRNA, miRNA, and methylation), we set the "method" parameter of the "getElites" function to "mad" to select the top 1,500 genes with the highest variation. Then, we set the "method" parameter to "cox" and combined clinical data to identify prognostic genes with a significance level of p < 0.05 for each data dimension. For binary variable gene mutation data, we first used the "oncoPrint" function from the maftools package to select the top 5,000 genes with the highest mutation frequency. Then, we further filtered based on mutation frequency by setting the "method" parameter to "freq" to identify the top 1% most frequently mutated genes. The results from these five dimensions were included in our study for further analysis. After the initial feature selection, we determined the optimal number of clusters for the study. Therefore, we used the "getClustNum" function from the MOVICS package, which integrates cluster prediction index (CPI), gap statistics, and Silhouette scores to estimate the number of subgroups. We ultimately decided to divide the samples into three subtypes. Subsequently, we applied the "getMOIC" function for cluster analysis. We used 10 clustering algorithms (CIMLR, ConsensusClustering, SNF, iClusterBayes, PINSPlus, moCluster, NEMO, IntNMF, COCA, and LRA) as input for the "methodslist" parameter and used default parameters provided by the MOVICS package. As a result, we obtained the clustering results for each method. After calculating the clustering results for the 10 methods, we integrated the results of different algorithms based on the concept of consensus clustering using the "getConsensusMOIC" function to improve the robustness of the clustering.

**Risk Features Generated by Machine Learning-Based Integrated Methods**

To develop a model called consensus machine learning-driven signature (CMLS) with high accuracy and stability, we integrated 10 machine learning algorithms and 101 algorithm combinations. The integrated algorithms included Random Survival Forest (RSF), Elastic Net (Enet), Lasso, Ridge, Stepwise Cox, CoxBoost, Cox Partial Least Squares Regression (plsRcox), Supervised Principal Components (SuperPC), Generalized Boosted Regression Models (GBM), and Survival Support Vector Machine (survival-SVM). The signature generation procedure was as follows: (a) Identification of prognostic genes using univariate Cox regression analysis in the TCGA-THCA dataset (as mentioned in the previous step); (b) Fitting of the prognostic genes with 101 algorithm combinations to build predictive models based on the leave-one-out cross-validation (LOOCV) framework in the TCGA-Train cohort; (c) Detection of all models in two validation datasets (TCGA-Test and TCGA-Entire); (d) Calculation of Harrell's concordance index (C-index) for each model in all validation datasets and selection of the model with the highest average C-index as the best model.
